# Mechanotransduction via TRPV4 regulates inflammation and differentiation in fetal mouse distal lung epithelial cells

**DOI:** 10.1186/s12931-015-0224-4

**Published:** 2015-05-27

**Authors:** Pritha S. Nayak, Yulian Wang, Tanbir Najrana, Lauren M. Priolo, Mayra Rios, Sunil K. Shaw, Juan Sanchez-Esteban

**Affiliations:** Department of Pediatrics, Women and Infants Hospital of Rhode Island and the Warren Alpert Medical School of Brown University, 101 Dudley Street, Providence, RI 02905 USA

**Keywords:** TRPV4, Lung, Fetal epithelial cells, Mechanotransduction, Inflammation, Differentiation

## Abstract

**Background:**

Mechanical ventilation plays a central role in the injury of premature lungs. However, the mechanisms by which mechanical signals trigger an inflammatory cascade to promote lung injury are not well-characterized. Transient receptor potential vanilloid 4 (TRPV4), a calcium-permeable mechanoreceptor channel has been shown to be a major determinant of ventilator-induced acute lung injury in adult models. However, the role of these channels as modulators of inflammation in immature lungs is unknown. In this study, we tested the hypothesis that TRPV4 channels are important mechanotransducers in fetal lung injury.

**Methods:**

Expression of TRPV4 in the mouse fetal lung was investigated by immunohistochemistry, Western blot and qRT-PCR. Isolated fetal epithelial cells were exposed to mechanical stimulation using the Flexcell Strain Unit and inflammation and differentiation were analyzed by ELISA and SP-C mRNA, respectively.

**Results:**

TRPV4 is developmentally regulated in the fetal mouse lung; it is expressed in the lung epithelium and increases with advanced gestation. In contrast, in isolated epithelial cells, TRPV4 expression is maximal at E17-E18 of gestation. Mechanical stretch increases TRPV4 in isolated fetal epithelial cells only during the canalicular stage of lung development. Using the TRPV4 agonist GSK1016790A, the antagonist HC-067047, and the cytokine IL-6 as a marker of inflammation, we observed that TRPV4 regulates release of IL-6 via p38 and ERK pathways. Interestingly, stretch-induced differentiation of fetal epithelial cells was also modulated by TRPV4.

**Conclusion:**

These studies demonstrate that TRPV4 may play an important role in the transduction of mechanical signals in the fetal lung epithelium by modulating not only inflammation but also the differentiation of fetal epithelial cells.

## Background

Mechanical forces generated in utero by repetitive breathing movements and by fluid distension are essential to mammalian lung development [1–3]. Throughout gestation, the lung epithelium actively secretes fluid creating a constant distension pressure of around 2.5 mmHg in the potential airspaces [4]. In addition, the fetus makes episodic breathing movements generating around 5 % changes in the distal lung surface area [5]. It is well established that both tonic hydrostatic distension and cyclic mechanical deformation provide physical signals necessary for normal fetal lung development [3, 6–8]. Paradoxically, many premature infants born with underdeveloped lungs are exposed to excessive, non-physiological levels of stretch. This may result in ventilator-induced lung injury which plays an important role in the pathogenesis of bronchopulmonary dysplasia (BPD), a chronic inflammatory lung disease with serious short- and long-term morbidities [9]. Excessive stretch of the lung by mechanical ventilation can disrupt the integrity of the alveolar-capillary barrier, resulting in interstitial and alveolar edema. Neutrophils and macrophages recruited to the lung can then trigger and amplify an injury response by releasing cytokines and other inflammatory mediators [10]. In addition, distal lung parenchyma cells can be directly exposed to overstretch and injury secondary to mechanical ventilation. It has been previously shown that mechanical stretch of type II cells releases pro-inflammatory cytokines [11–13] and are an important source of chemokines that orchestrate leukocyte migration [14], supporting a role for parenchymal lung cells in the pathogenesis of BPD.

TRPV4 is a Ca^2+^-permeable cation channel known to play an important role in osmotic and mechanical sensing [15]. TRPV4 is widely expressed in mammalian tissues [16]. In the adult lung, TRPV4 has been found in the epithelial linings of trachea and airways, in the endothelium of bronchial and pulmonary artery and in the alveolar septum [17, 18]. TRPV4 channels control epithelial and endothelial barrier integrity in response to stretch or increased vascular pressure and are a major determinant of ventilator-induced acute lung injury [19, 20]. TRPV4 agonists produced blebs or break in the endothelial and epithelial layers of the alveolar wall and increased lung endothelial permeability [17]. Although TRPV4 blockade may represent a therapeutic approach to decrease pulmonary edema in adult lung exposed to mechanical ventilation, whether similar strategy is also applicable to underdeveloped lungs is unknown. This distinction is critical given that the anatomy, histology and function of the lung are different in the fetus versus the adult. For example, the distal lung epithelium during the canalicular stage of lung development is lined by undifferentiated cuboidal cells with a phenotype intermediate between type I and type II epithelial cells [21, 22].

Therefore, the purpose of these investigations was to study the role of TRPV4 in the injury of the fetal lung during the canalicular stage of lung development, a period of time in gestation where many extreme premature infants are born and exposed to mechanical injury. We used an in vitro system where isolated distal epithelial cells were exposed to mechanical stretch mimicking lung injury. We found that in addition to regulate inflammation, TRPV4 channels participate in the differentiation of fetal epithelial cells mediated by mechanical stretch.

## Methods

### Cell isolation and stretch protocol

This study was carried out in strict accordance with the recommendations in the Guide for the Care and Use of Laboratory Animals of the National Institutes of Health. The protocol was approved by the Lifespan Institutional Animal Care and Use Committee, Providence, RI (Protocol # 0031–13). Fetal mouse lungs were obtained from timed-pregnant C57BL6 at different times in gestation (E17-E19) and distal lung epithelial cells were isolated as previously described [23]. Briefly, after collagenase or dispase digestion, cell suspensions were sequentially filtered through 100-, 30-, and 20-μm nylon meshes using screen cups (Sigma). Clumped nonfiltered cells from the 30- and 20-μm nylon meshes were collected after several washes with DMEM to facilitate the filtration of nonepithelial cells. Further epithelial cell purification was achieved by incubating the cells in 75-cm^2^ flasks for 30 min. Non-adherent cells were collected and cultured overnight in 75-cm^2^ flasks containing serum-free DMEM. After overnight culture, cells were harvested with 0.25 % (wt/vol) trypsin in 0.4 mM EDTA, and plated (around 50 % confluency) on Bioflex multiwell plates (Flexcell International, Hillsborough, NC) precoated with fibronectin (1.5 μg/cm^2^). Monolayers were maintained in culture for 1–2 days until they were approximately 80 % confluents and then were mounted in a Flexcell FX-4000 Strain Unit (Flexcell International). Equibiaxial cyclical strain regimen of 20 % was applied at intervals of 40 cycles/min for different lengths of time. This regimen, which roughly corresponds to a lung inflation of 80 % of total lung capacity in adult rats [24–26], was chosen to mimic lung cells injury. To simulate physiologic stretch, monolayers coated on laminin substrates (2 μg/cm^2^) were exposed to 5 % intermittent strain [27]. Cells were grown on nonstretched membranes in parallel and were treated in an identical manner to serve as controls. The rational to use laminin for the physiologic stretch experiments is based on the highest level of epithelial differentiation observed with this substrate [28]. In contrast, in monolayers exposed to injurious stretch, cells detached from the membranes in laminin-coated substrates but not in fibronectin.

### Real-time PCR (qRT-PCR)

Total RNA was isolated as previously described [29] and purified further using the Turbo DNA-free kit (Ambion). One microgram of total RNA was reverse-transcribed into cDNA using the iScript™ cDNA Synthesis Kit (Bio-Rad) according to the manufacturer’s instructions. Pre-designed TaqMan® primers were purchased from Assays-on-Demand™ Gene Expression Products (Life technology).

The following primers were used: TRPV4 (cat #: Mm00499025_m1), SP-C (cat #: Mm00488144_m1). To amplify the cDNA by qRT-PCR, 4 μl of the resulting cDNA were added to a mixture of 10 μl of TaqMan Gene Expression Master Mix (Life technology) and Assays-on-Demand™ Gene Expression Assay Mix containing forward and reverse primers and TaqMan labeled probe (Life technology). Standard curves were generated for each primer set and housekeeping gene GAPDH (cat #: Mm99999915_g1). Linear regression revealed efficiencies between 96 and 99 %. Therefore, fold expressions of stretched samples relative to controls were calculated using the ∆∆C_T_ method for relative quantification (RQ) as previously described [30]. Samples were normalized to GAPDH. The reactions were performed in a 7500 Fast Real-Time PCR System (Applied Biosystems) with the following parameters: 50 °C – 2 min, 95 °C – 10 min, and 45 cycles of 95 °C – 15 s, 60 °C – 1 min. All assays were performed in triplicate.

### Western blot analysis

Lung tissue was isolated, frozen in liquid nitrogen and stored at −80 °C until analysis. For protein extraction, tissue samples were minced and sonicated, and similar to cell monolayers, samples were lysed in RIPA buffer containing protease inhibitors. Lysates were centrifuged and total protein contents were determined by the bicinchoninic acid method. Equal amount of protein lysate samples (20 μg) were fractionated by NU-PAGE Bis-Tris (4–12 %) gel electrophoresis (Novex, San Diego, CA) and transferred to polyvinylidene difluoride membranes. Blots were hybridized with antibody to TRPV4 [1:100] (cat # ACC-034, Alomone, Jerusalem, Israel), phospho (p)-JNK, p-PLA_2_, p-p38 or p-ERK [1:100] (all from Cell Signaling Technology, Danvers, MA). Goat anti-rabbit secondary antibodies [1:10,000] were conjugated with horseradish peroxidase; blots were developed with an enhanced chemiluminescence (ECL) detection assay (Amersham Pharmacia Biotech, Piscataway, NJ). Membranes were then stripped and reprobed with antibodies to vinculin, GAPDH, total (t)-JNK, t-PLA_2_, t-p38 or t-ERK [1:100] (to control for protein loading) and processed as described before. The intensity of the bands was analyzed by densitometry.

### Concentration of IL-6 in the supernatant

E17 epithelial cells were exposed to 20 % cyclic stretch for 48 h. After experiments, the cell culture medium was collected, centrifugated to remove cell debris and stored at −80 °C before analysis. Cell monolayers from the BioFlex plates were lysed with ice-cold RIPA buffer (150 mM NaCl, 100 mM Tris base, pH 7.5, 1 % deoxycholate, 0.1 % SDS, 1 % Triton X-100, 3.5 mM Na_3_VO_4_, 2 mM PMSF, 50 mM NaF, 100 mM sodium pyrophosphate) with protease inhibitors (10 μg/ml leupeptin, 10 μg/ml aprotinin, 143.5 μM aminoethyl benzenesulfonyl fluoride). Lysates were centrifuged and total protein content was determined by the bicinchoninic acid method. IL-6 concentration in the supernatant was measured using a commercial ELISA kit (Enzo Life Sciences, Farmingdale, NY, cat # ADI-900-045) according to the manufacture’s recommendations. The optical density was determined photometrically at 450 nm using the ELISA plate reader EL_x_800 (Bio-Tek Instruments). Results were normalized to the cell lysate concentration in each sample as a representation of the number of cells added to the wells.

### Immunohistochemistry

After isolation, fetal lung tissue was fixed by immersion in 10 % formalin. Sections (5 μm) were processed for immunohistochemistry using a rabbit anti-TRPV4 polyclonal antibody (Alomone) stained with diaminobenzidine and counterstained with hematoxylin. Briefly, tissue sections mounted on the slides were deparaffinized and rehydrated with xylene and ethanol followed by heat-induced antigen retrieval using sodium citrate buffer. Slides were then blocked with 3 % BSA in PBS-T for 1 h at room temperature and incubated with anti-TRPV4 antibody [1:100] overnight at 4 °C. The next day, slides were incubated with biotinylated goat-anti-rabbit HRP secondary antibody [1:200] for 1 h at room temperature, followed by a hydrogen peroxidase block, diaminobenzidine staining and counterstaining with hematoxylin. Slides were then allowed to air dry and hydrosoluble mounting media was added and mounted on the coverslip until analysis.

### Statistical analysis

Results are expressed as means ± SEM from at least 3 experiments, using different litters for each experiment. Data were analyzed with ANOVA followed by post hoc tests, and Instat 3.0 (GraphPad Software, San Diego, CA) was used for statistical analysis; *P* < 0.05 was considered statistically significant.

## Results

### TRPV4 expression in the fetal lung is developmentally regulated

**Fig. 1:**
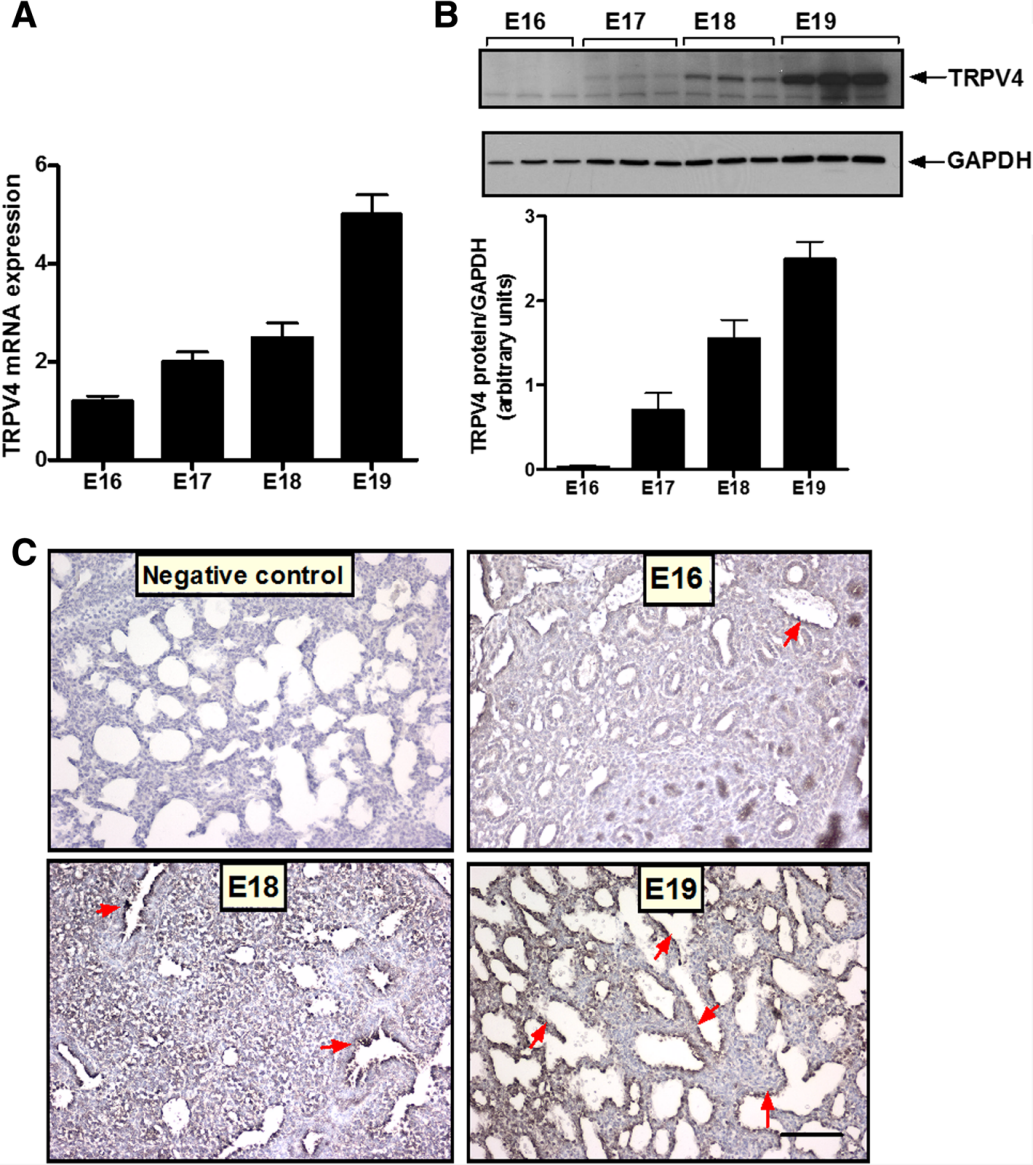
TRPV4 expression increases with gestation. **a** Fetal lung tissue was collected at different times in gestation, as shown. RNA was isolated, reversed-transcribed, and the cDNA products for TRPV4 were analyzed by quantitative RT-PCR. *N* = 3. **b** Fetal lung tissue was collected at different times in gestation and proteins extracted to assess TRPV4 expression by Western blot. The upper panel is a representative blot. Results were normalized to GAPDH to control for protein loading. *N* = 3. **c** Fetal lung tissue from different times in gestation was fixed in formalin. Sections were process by immunohistochemistry using rabbit anti-TRPV4 polyclonal antibody, stained with diaminobenzidine and counterstained with hematoxylin. Immunohistochemistry pictures show distribution of TRPV4 during fetal lung development (*arrows*). At E16 (*pseudoglandular stage*), TRPV4 was minimally expressed in the respiratory bronchioles. Later in gestation, TRPV4 immunostaining was more apparent in the distal epithelium. Bar, 20 μm

TRPV4 channels have been shown to respond to mechanical signals in different tissues [16]. To investigate whether these channels participate in mechanotransduction of the fetal lung, we analyzed first TRPV4 expression at different stages of lung development. As shown in Fig. 1a, TRPV4 mRNA expression increased with advanced gestation. Similarly, TRPV4 protein was barely detected during the pseudoglandular stage of lung development (E16) and progressively increased with gestation (Fig. 1b). Immunohistochemistry pictures (Fig. 1c) show minimal expression of TRPV4 in the respiratory bronchioles at E16. TRPV4 immunostaining was more apparent in the distal epithelium with advanced gestation. All together, these data demonstrate that TRPV4 expression in the fetal lung increases as gestation progresses.

### Mechanical stretch increases TRPV4 in isolated fetal epithelial cells only during the canalicular stage of lung development

**Fig. 2:**
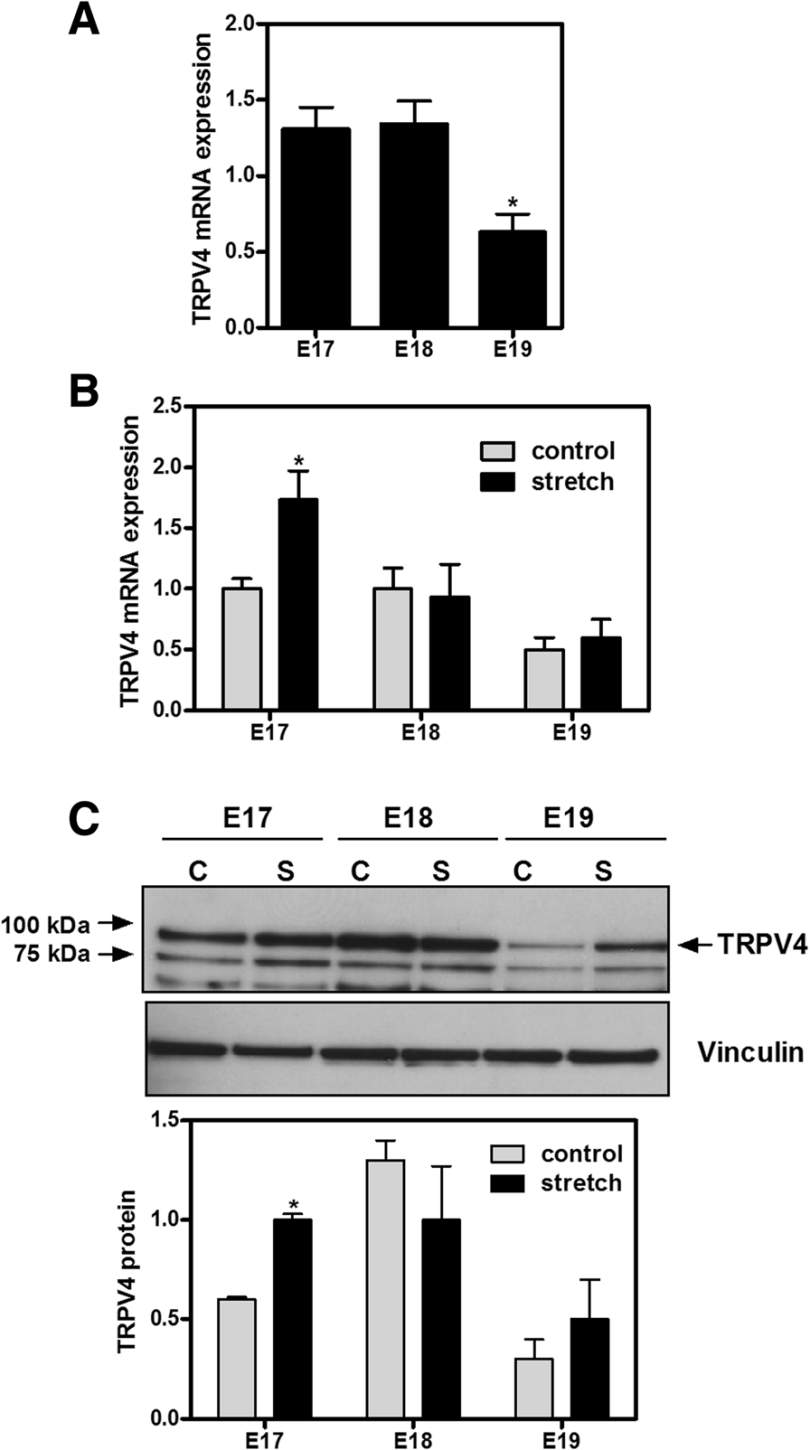
**T**RPV4 expression in isolated distal fetal epithelial cells and the effect of mechanical stretch. **a** E17-E19 fetal epithelial cells were isolated as described in *methods* and processed to analyze TRPV4 mRNA expression by qRT-PCR using the ∆∆C_T_ method for relative quantification (*n* = 4; *p < 0.02 vs E17 or E18, Tukey-Kramer Multiple Comparisons Test). **b** Fetal epithelial cells were isolated at E17-E19 of gestation and cultured on bioflex plates coated with fibronectin. Twenty four hours later, monolayers were exposed to 20 % cyclic stretch at 40 cycles/min for 24 h; unstretched samples were used as control. Samples were processed by qRT-PCR to assess TRPV4 mRNA expression (*n* = 4; *p < 0.05 vs E17 control, Tukey-Kramer Multiple Comparisons Test). **c** Fetal epithelial cells isolated from E17-E19 lungs were seeded on bioflex plates, as described above, and exposed to 20 % cyclic stretch for 24 h. Proteins were extracted and processed to determine TRPV4 protein abundance. The upper panel is a representative blot normalized to vinculin. Data in the lower panel are from 4 different experiments (*P < 0.01 vs E17 control, Tukey-Kramer Multiple Comparisons Test). *C* = control; *S* = stretch

Next we analyzed TRPV4 expression in isolated distal epithelial cells during the canalicular and saccular stages of lung development (E17-E19). In contrast to the whole lung, TRPV4 mRNA expression in isolated epithelial cells remains constant at E17 and E18 of gestation and decreased by 50 % at E19 (1.3 ± 0.14 vs 0.63 ± 0.12) (Fig. 2a). Similar results were observed on TRPV4 protein abundance (see control bars on Fig. 2c) except that TRPV4 protein expression seems to peak at E18. Mechanical stretch increased TRPV4 gene expression and protein abundance by 73 and 40 %, respectively, when compared to control, unstretched samples (1 ± 0.08 vs 1.73 ± 0.24 and 0.6 ± 0.01 vs 1 ± 0.03). However, this response was only present in cells isolated during the canalicular stage of lung development (E17) (Fig. 2b and c). All together, these data show that TRPV4 expression and response to mechanical forces in isolated fetal epithelial cells are gestational-age dependent.

### Release of the inflammatory cytokine IL-6 after mechanical injury is mediated via TRPV4

**Fig. 3:**
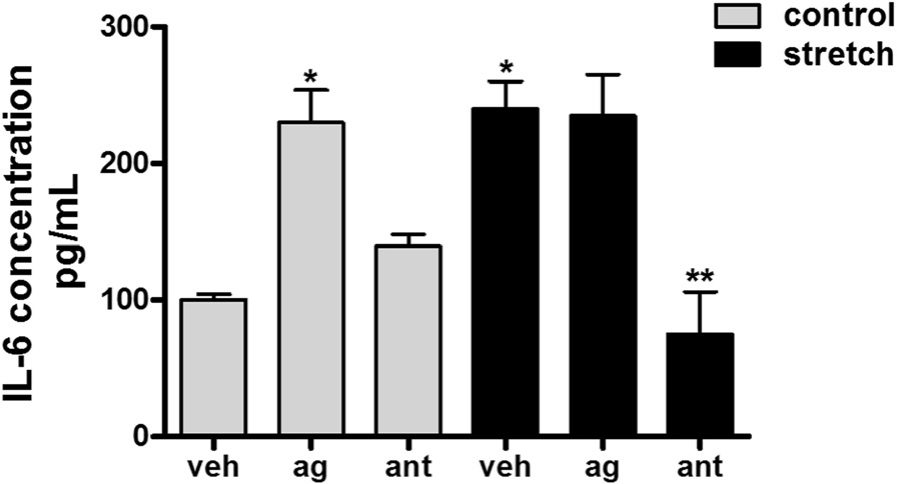
TRPV4 regulates stretch-induced release of IL-6. E17 epithelial cells were isolated and seeded on bioflex plates coated with fibronectin. 24 hours later, cells were exposed to 20 % cyclic stretch for 48 h in the presence or absence of the vehicle DMSO, the TRPV4 agonist GSK1016790A [100 nM] or TRPV4 antagonist HC-067047 [1 μM]. Unstretched cells served as controls. Supernatants were collected and processed to assess IL-6 concentrations by ELISA, as described in methods. Values are mean ± SEM from 5 different experiments. Results were normalized to the cell lysate protein concentrations. *p < 0.05 vs control vehicle; **p < 0.01 vs stretch vehicle. Tukey-Kramer Multiple Comparisons Test. Veh = vehicle, DMSO; ag = agonist GSK1016790A; ant = antagonist HC-067047

Previous studies from our laboratory have demonstrated that mechanical injury of fetal epithelial cells releases pro-inflammatory cytokines [12, 31]. Given the role of TRPV4 in regulating inflammation in other systems and the activation of TRPV4 in fetal epithelial cells, we investigated whether this channel participates in fetal epithelial cell inflammation mediated by stretch. For these experiments, we used the pro-inflammatory cytokine IL-6 as a marker of inflammation. IL-6 is well-known to play a key role in the mechanical injury of premature lungs and shown to be increased by stretch [31, 32]. Fetal epithelial cells were exposed to 20 % cyclic stretch for 48 h in the presence or absence of TRPV4 agonist/antagonist. Figure 3 shows and as expected, injurious stretch increased release of IL-6 by 2.4-fold (100 ± 4.1 vs 240 ± 20). Interestingly, the addition of the TRPV4 agonist GSK1016790A [100 nM] [33] was sufficient to increase release of IL-6 in control samples (100 ± 4.1 vs 230 ± 24). Mechanical stretch, in the presence of the TRPV4 agonist, did not further increase the release of IL-6 when compared to control agonist or vehicle stretch. In contrast, blockade of this channel with HC-067047 [1 μM] [34] significantly decreased stretch-induced release of IL-6 by 70 % when compared to vehicle stretch (240 ± 20 vs 75 ± 31). These data strongly suggest that TRPV4 modulates the release of IL-6 in fetal epithelial cells exposed to injurious stretch.

### Activation of IL-6 by TRPV4 is mediated via p38 and ERK pathways

**Fig. 4:**
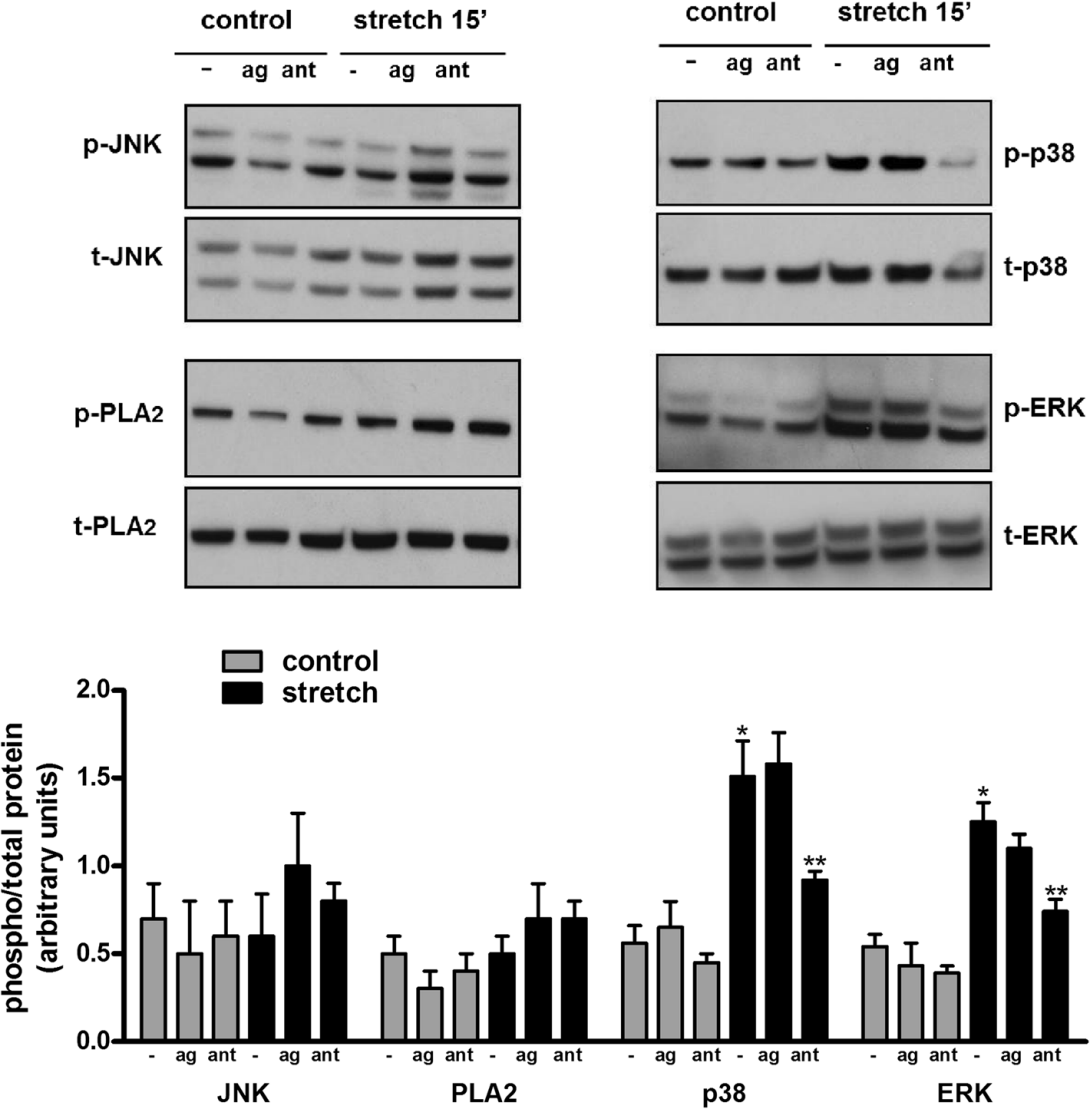
Stretch-induced activation of TRPV4 is mediated via p38 and ERK pathways. E17 distal lung epithelial cells were isolated and seeded on plates coated with fibronectin. The following day, monolayers were exposed to 20 % cyclic stretch at 40 cycles/min for 15 min in the presence or absence of the vehicle DMSO, the TRPV4 agonist GSK1016790A [100 nM] or TRPV4 antagonist HC-067047 [1 μM]. The level of activation of the indicated proteins in the cell lysate was evaluated by Western blot using phospho-specific antibodies. Blots were then stripped and reprobed with total antibodies to control for protein loading. Upper panels are representative Western blots. Data in the lower panel are from 5 different experiments. *p < 0.05 vs negative control; **p < 0.01 vs negative stretch. Tukey-Kramer Multiple Comparisons Test

**Fig. 5:**
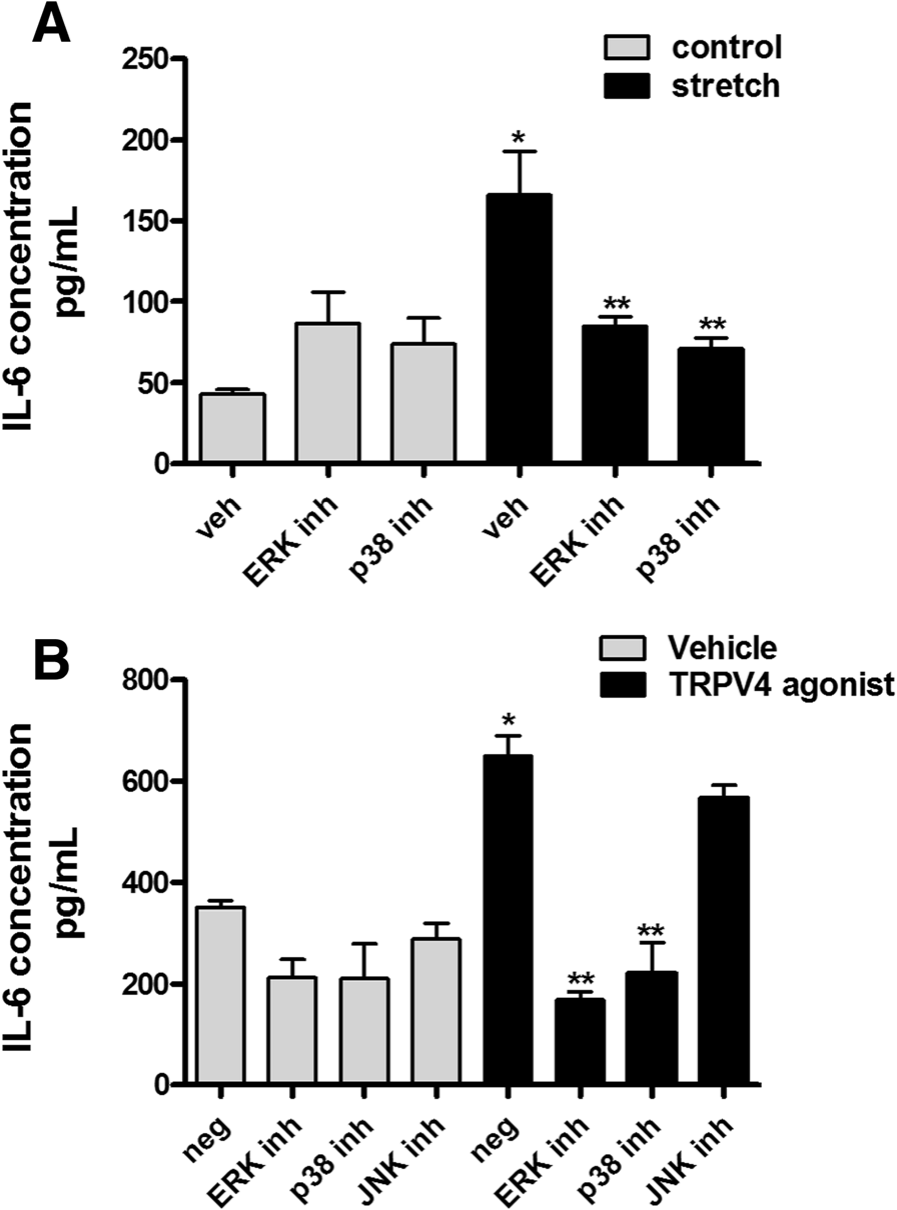
Release of IL-6 by stretch or TRPV4 agonist is regulated via ERK and p38 pathways. **a** E17 epithelial cells seeded on bioflex plates coated with fibronectin were preincubated for 30 min with the ERK pathway inhibitor U0126 [20 μM] or the p38 inhibitor SB203580 [20 μM] and then exposed to 20 % cyclic stretch for 48 h. Supernatants were collected and the concentration of IL-6 was analyzed by ELISA, as described in methods. Data are normalized to the cell lysate concentrations. *N* = 4; *p < 0.01 vs vehicle control; **p < 0.01 vs vehicle stretch. Tukey-Kramer Multiple Comparisons Test. **b** E17 epithelial cells seeded on fibronectin-coated plates were incubated for 48 h with the ERK pathway inhibitor U0126 [20 μM], the p38 inhibitor SB203580 [20 μM] or the JNK inhibitor SP600125 [20 μM], in the presence of DMSO (vehicle) or the TRPV4 agonist GSK1016790A [100 nM]. Supernatants were collected and the concentration of IL-6 was analyzed by ELISA, as described above. *N* = 3; *p < 0.05 vs negative vehicle; **p < 0.01 vs TRPV4 agonist. Tukey-Kramer Multiple Comparisons Test

We then investigated potential signaling pathways regulating release of IL-6 via TRPV4. For these experiments fetal epithelial cells were exposed to 20 % stretch in the presence or absence of agonist/antagonist of TRPV4. Figure 4 demonstrates that neither JNK nor PLA2 were activated by stretch or their phosphorylation levels affected by TRPV4 modulators. In contrast, p38 and ERK pathways were stimulated after 15 min of cyclic stretch by 3-fold and 2-fold, respectively (0.56 ± 0.1 vs 1.5 ± 0.2 and 0.54 ± 0.07 vs 1.25 ± 0.1). Incubation of epithelial cells with the TRPV4 blocker HC-067047 [1 μM] decreased stretch-induced activation of both pathways by 40 % (1.5 ± 0.2 vs 0.92 ± 0.05 and 1.25 ± 0.1 vs 0.74 ± 0.07). These studies indicate that activation of these two pathways by stretch is partially mediated via TRPV4. To further investigate the role of these two pathways in mechanical injury and specifically in the release of IL-6, isolated fetal epithelial cells were exposed to 20 % stretch in the presence of the ERK inhibitor U0126 [20 μM] or p38 inhibitor SB203580 [20 μM] [35] and release of IL-6 into the supernatant was investigated by ELISA. As shown in Fig. 5a, mechanical injury released IL-6 by 4.7-fold when compared to unstretched samples. Incubation with ERK or p38 inhibitors did not affect IL-6 release under static conditions but significantly decreased IL-6 release after mechanical stretch by 60 % and 75 %, respectively (4.7 ± 0.5 vs 1.8 ± 0.2 and 4.7 ± 0.5 vs 1.17 ± 0.17). We also investigated whether stimulation of IL-6 by TRPV4 agonist (Fig. 3, bar 2) was mediated via MAPK pathways. Data in Fig. 5b show that both ERK and p38 inhibitors decrease IL-6 release by 40 %. In contrast, blockade of JNK pathways has not effect. All together these results indicate that mechanical injury activates ERK and p38 and both pathways participate in the release of IL-6 via TRPV4.

### Stretch-induced fetal epithelial cell differentiation is mediated via TRPV4

**Fig. 6:**
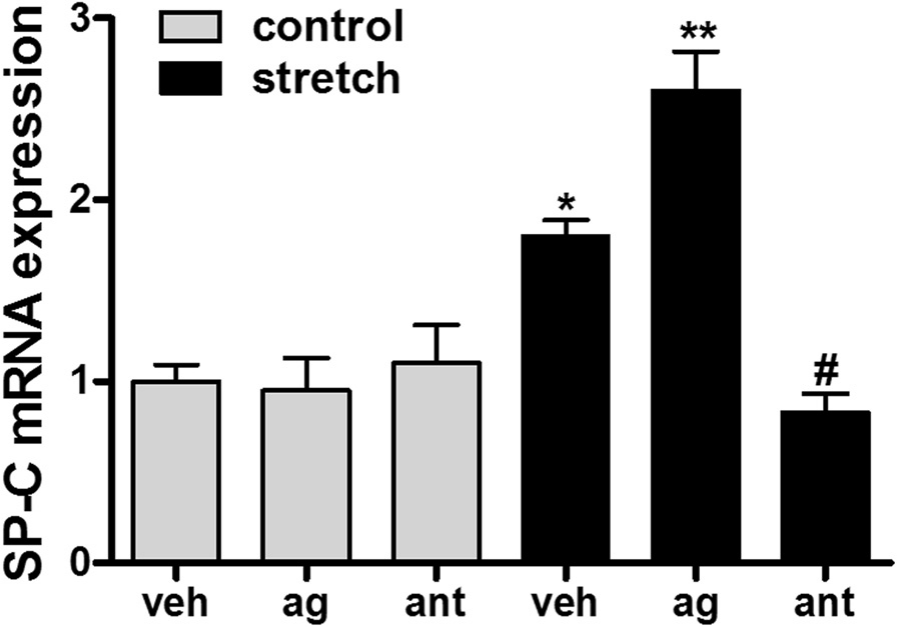
Stretch-induced fetal epithelial cell differentiation is mediated via TRPV4. E17 epithelial cells were seeded on bioflex plates coated with laminin and then exposed to a physiologic 5 % cyclic stretch at 40 cycles/min for 24 h, in the presence or not of the TRPV4 agonist GSK1016790A [100 nM] or TRPV4 antagonist HC-067047 [1 μM]. Unstretched cells served as controls. RNA was extracted, as described in methods, and processed to assess SP-C mRNA abundance by qRT-PCR. Results are from 5 separate experiments. *P < 0.05 vs vehicle control; **P < 0.05 vs vehicle stretch; #*P* < 0.01 vs vehicle stretch. Tukey-Kramer Multiple Comparisons Test

We finally addressed whether TRPV4 participates in stretch-induced differentiation of fetal epithelial cells. Previous studies from our laboratory have shown that 5 % cyclic stretch for 24 h upregulates surfactant protein-C (SP-C), a specific marker of type II cell differentiation [27]. To investigate whether this channel participates in the differentiation of epithelial cells mediated by stretch, monolayers were exposed to a physiologic 5 % stretch in the presence of specific TRPV4 agonist or antagonist. Our data in Fig. 6 show and as expected, mechanical stretch upregulated SP-C when compared to vehicle, control samples. The addition of the TRPV4 agonist GSK1016790A to the culture media did not affect SP-C in unstretched samples but increased SP-C mRNA by 44 % when compared to stretch samples without agonist (1.8 ± 0.09 vs 2.6 ± 0.22). In contrast, incubation of cells with the antagonist HC-067047 decreased SP-C mRNA by 54 % when compared to stretch vehicle (1.8 ± 0.09 vs 0.83 ± 0.1). The data suggest that TRPV4 participates in the differentiation of fetal epithelial cells mediated by stretch.

## Discussion

Many premature infants born with underdeveloped lungs develop BPD, a chronic lung disease with potential serious long-term complications. Injury mediated by mechanical ventilation plays a central role in the pathogenesis of BPD. However, the mechanisms by which mechanical injury promotes lung inflammation are not fully-elucidated. Although TRPV4 channels have been shown to regulate pulmonary edema and inflammation in adult lungs, the role of these channels in underdeveloped lungs is unknown. The main findings of this study are: 1) TRPV4 is expressed in the fetal lung. 2) TRPV4 may play an important role in the transduction of mechanical signals in the distal epithelium by modulating inflammation via p38 and ERK pathways. 3) TRPV4 also participates in stretch-mediated differentiation of type II epithelial cells.

Calcium signaling is important for different aspects of fetal lung development, such as branching morphogenesis [36] and surfactant release [15]. Since mechanical forces are critical for normal lung development and TRPV4 channels facilitates cellular entrance of calcium in response to mechanical signals [16], it was not a surprise to find that TRPV4 is expressed in the fetal lung, and specifically in the bronchial and distal epithelium. These results are consistent with studies in the adult mouse lung, where TRPV4 was detected in the bronchiolar epithelium and alveolar septal wall [17]. Although immunohistochemistry images show TRPV4 staining only in the epithelium, we cannot rule out that TRPV4 is also expressed in the endothelium, as demonstrated by electron microscopy in adult rodents [17]. The presence of TRPV4 in the epithelium of the distal lung was also corroborated in isolated epithelial cells, where TRPV4 reached maximal expression during the canalicular/early saccular stages of lung development. Previous investigations have also shown the presence of TRPV channels in alveolar type II cells and found to be critical as a mediator of strain-induced calcium entry into the cells [37]. We tried to demonstrate that mechanical strain increases uptake of calcium via TRPV4. However, we found an erratic and inconsistent response to stretch. It might be possible that the response of epithelial cells to stretch is very fast and unable to be detected using our experimental system (StageFlexer Jr, Flexcell International, Burlington, NC). It has been demonstrated for example, using a micromanipulator and micro-spatula, that strain-induced calcium entry via TRPV2 in alveolar type II cells is very fast (<30 ms) [37]. Another explanation could be the amplitude of strain and cell-cell contacts, both of them affecting strain-induced calcium signal [38]. Despite the limitations of our experimental system to directly demonstrate that mechanical strain uptakes calcium via TRPV4 and based on our findings, we speculate that TRPV4 may be relevant to calcium signaling in the epithelium of the developing lung.

In adult lungs, the surface of the alveolus is mainly covered by type I epithelial cells and studies in adult mouse lungs have shown that TRPV4 is expressed in the capillary of septal wall and in alveolar type I cells. TRPV4 agonists caused disruption of the alveolar epithelium with detachment of alveolar type I cells from the basement membrane, suggesting that TRPV4 plays a key role in the alveolar-capillary barrier [17]. However, many premature infants are born during the canalicular stage of lung development and the distal lung epithelium is still covered by undifferentiated cuboidal cells [21, 22]. Furthermore, these premature lungs are exposed to injury secondary to mechanical ventilation causing pulmonary edema and inflammation. TRPV4 has previously been demonstrated to be expressed in respiratory epithelial cells [28, 39, 40] and another cation channel, TRPA1, was present in human pulmonary alveolar epithelial cell line A549 cells [41]. However, whether these undifferentiated distal epithelial cells express TRPV4 and the response to mechanical injury were unknown. Our data clearly demonstrate not only that TRPV4 is present in these cells but also that the expression level responds to mechanical signals. In addition, we found that the response to mechanical stimulation was gestational-age dependent, being the canalicular stage of lung development the period of gestation more sensitive to mechanical stimuli, as previously shown [27, 42]. TRPV4 was found to decrease with advanced gestation in isolated epithelial cells. The apparent contraction with the increased expression in the whole lung could be explain by a shift in the phenotype of the cells lining the epithelium from cuboidal undifferentiated to type I epithelial cells, whereas the population of type II cells will be decreased and localized only in the corners of the alveoli.

In addition to regulate the integrity of the lung alveolar capillary endothelium [43–45] and to maintain the airway epithelial barrier function [46], TRPV4 has been recently implied as a key mediator of inflammation. TRPV4 inhibitors have potent anti-inflammatory effects by limiting neutrophil and macrophage infiltration, and blunting pro-inflammatory cytokine and chemokine production [47]. However, the mechanisms underlying these effects are not well-understood. One possibility is that TRPV4 inhibitors act primarily on endothelial and epithelial cells, not only preventing changes in barrier function, but also blocking other Ca2+ dependent processes, such as the release of cytokines and adhesion molecules or the facilitation of neutrophil transit [48]. Our data support this hypothesis and found that TRPV4 regulates release of IL-6 in fetal epithelial cells exposed to injurious stretch. Even the administration of a TRPV4 agonist, in the absence of stretch, was sufficient to increase the release of IL-6. Therefore, our investigations provide novel observations that distal fetal epithelial cells are an important source of inflammatory cytokines, as previously shown in differentiated alveolar type II epithelial cells [11] and their release is modulated by TRPV4. All together, these studies suggest that TRPV4 may play a key role in modulating inflammation in the distal epithelium of premature lungs exposed to mechanical injury.

We investigated next the signaling pathways by which mechanical stretch regulates release of IL-6 via TRPV4. We found that neither JNK nor PLA_2_ were activated by mechanical stretch or their phosphorylation levels affected by activation/inhibition of TRPV4 channels. In contrast, mechanical stretch activated ERK and p38 pathways and blockade of TRPV4 decreased stretch-mediated activation of these proteins. These data indicate that activation of these pathways is at least partially mediated via TRPV4. The TRPV4 agonist did not activate p38 or ERK as it would have been expected. However, the addition of GSK101 to HeLa cells led to an early rapid activation of the TRPV4 channel (within seconds) followed by a quick decrease, indicating desensitization of the channel [49]. Since, activation of the MAPK signaling pathways were evaluated after 15 min of stretch, it is conceivable that phosphorylation of p38 and ERK induced by the TRPV4 agonist GSK101 might have happened earlier and by 15 min returned to the baseline. Moreover, release of IL-6 by stretch, which we found to be modulated by TRPV4, is also mediated via p38 and ERK pathways. Therefore, all together these studies provide indirect evidences that these two pathways participate in stretch-induced release of IL-6 via TRPV4. These results are consistent with previous studies in cultured chondrocytes showing that activation of TRPV4 by osmotic stress was mediated via ERK and p38 pathways [50]. In addition, release of inflammatory cytokines in corneal epithelium after activation of TRPV1 was also mediated through MAPK signaling [51]. Therefore, both pathways seem to be important downstream activators of TRPV4. However, the specific contribution of these signaling pathways to the regulation of inflammation needs further investigations.

As discussed in the background section, low level of mechanical stretch (“physiologic” stretch) is critical for normal lung development [1–3]. Given that calcium signaling is also important for lung development [36, 52], we studied whether TRPV4 channels participate in differentiation of fetal epithelial cells mediated by physiologic stretch using an experimental system and a marker of type II cell differentiation previously used in our laboratory [8, 23, 27, 29, 53]. We found that activation of TRPV4 potentiated stretch-mediated differentiation of fetal epithelial cells whereas inhibition of this channel decreased stretch-induced differentiation. However, TRPV4 knockout mice do not exhibit any baseline lung pathology, and are thought to have grossly normal lung development [54]. This apparent discrepancy could be explained by compensatory mechanisms regulating stretch-mediated differentiation of fetal lung epithelial cells. Therefore, these results indicate that in addition to regulate inflammation, TRPV4 also participates in important physiological mechanical signals for the differentiation of fetal epithelial cells. As recently discussed in an excellent editorial review [55], TRPV4 blockade may represent a double-edged sword, where the therapeutic benefits of TRPV4 inhibition have to be carefully weighed against potential adverse effects.

## Conclusions

Our data provide the first evidence that TRPV4 channels are present in the epithelium of the fetal lung and could play important roles not only in modulating the release of inflammatory cytokines after mechanical injury, but also in the differentiation of distal fetal lung epithelial cells. Although TRPV4 inhibitors could be a promising therapeutic strategy for the treatment of BPD in premature infants, more studies are necessaries to carefully evaluate the potential side effects related to their contribution to calcium signaling in normal fetal lung development. Specifically, genetically modified mice in the context of lung injury mediated by neonatal mechanical ventilation or hyperoxia could be effective in vivo models to further investigate the role of TRPV4 in mediating inflammation of premature lungs.

## Abbreviations

BPD: Bronchopulmonary dysplasia
SP-C: Surfactant protein-C
TRPV: Transient receptor potential vanilloid 4
